# The new biology: beyond the Modern Synthesis

**DOI:** 10.1186/1745-6150-2-30

**Published:** 2007-11-24

**Authors:** Michael R Rose, Todd H Oakley

**Affiliations:** 1 Department of Ecology and Evolutionary Biology, University of California, Irvine, CA, 92697-2525 USA; 2 Department of Ecology, Evolution, and Marine Biology, University of California, Santa Barbara, CA 93106-9610 USA

## Abstract

**Background:**

The last third of the 20^th ^Century featured an accumulation of research findings that severely challenged the assumptions of the "Modern Synthesis" which provided the foundations for most biological research during that century. The foundations of that "Modernist" biology had thus largely crumbled by the start of the 21^st ^Century. This in turn raises the question of foundations for biology in the 21^st ^Century.

**Conclusion:**

Like the physical sciences in the first half of the 20^th ^Century, biology at the start of the 21^st ^Century is achieving a substantive maturity of theory, experimental tools, and fundamental findings thanks to relatively secure foundations in genomics. Genomics has also forced biologists to connect evolutionary and molecular biology, because these formerly Balkanized disciplines have been brought together as actors on the genomic stage. Biologists are now addressing the evolution of genetic systems using more than the concepts of population biology alone, and the problems of cell biology using more than the tools of biochemistry and molecular biology alone. It is becoming increasingly clear that solutions to such basic problems as aging, sex, development, and genome size potentially involve elements of biological science at every level of organization, from molecule to population. The new biology knits together genomics, bioinformatics, evolutionary genetics, and other such general-purpose tools to supply novel explanations for the paradoxes that undermined Modernist biology.

**Open Peer Reviewers:**

This article was reviewed by W.F. Doolittle, E.V. Koonin, and J.M. Logsdon. For the full reviews, please go to the Reviewers' Comments section.

## Background

### The first two biological syntheses

Biology has been re-integrated twice already, first by Darwin in 1859 and then during the "Modern Synthesis" of the 1920s and 1930s. In both cases, the success of these syntheses rested in part on ignorance. Charles Darwin could reasonably integrate biology in the 19^th ^Century on a relatively elegant evolutionary foundation partly because a great deal was not yet known about cellular and biochemical machinery. This is not to say that Darwin could not have integrated the findings of 20^th ^Century cell biologists and geneticists into his theory; he simply didn't have the opportunity to do so because the data were not yet available. He did the best that he could with the scientific material of biology that was widely available in his day, and he almost single-handedly effected the first integration of the biological sciences.

Nevertheless, Darwin's synthesis was seriously and legitimately questioned in the first years of the 20^th ^Century [1] particularly due to the impact of new findings in genetics and cell biology. There was too much detail that was unknown during Darwin's time, most notably a workable theory for the inheritance of quantitative traits, for Darwin's synthesis to last without considerable reformulation. But the disintegration of the first attempt at scientific biology would naturally enough pave the way for the next, as Fisher, Haldane, Wright, Dobzhansky, Mayr, Simpson, and Stebbins, among many others, integrated genetics, paleontology, systematics, and cytology within a new, expanded, structure for biological thought that is often referred to as "The Modern Synthesis". After its genesis, this Modern Synthesis provided useful foundations for biological thought for the middle part of the 20^th ^Century.

Like Darwin's synthesis, the form of the Modern Synthesis was shaped in part by ignorance of important features of life that were at the time unknown to science. Specifically, the molecular biology of the cell remained largely unknown. During the construction of The Modern Synthesis, molecular biology was in its infancy. The biochemistry and molecular biology of the gene had not yet been worked out, leaving evolutionary geneticists free to imagine that genomes were orderly libraries of stable hereditary information strongly shaped by natural selection. And so they did, in most cases. This made it possible for them to use simple models to supply excellent solutions to such important and previously unsolved problems as the inheritance of quantitative variation, the action of natural selection on Mendelian variation, the role of chromosome rearrangements in speciation, and so on. We are great fans of the achievements of the Modern Synthesis, particularly its clarity, its mathematically explicit foundations, and its capacity to make sense of a broad range of biological phenomena. In this respect, the Modern Synthesis shares many features with Newtonian physics.

Nonetheless, the view of life that most biologists had from 1935 to 1965 was highly simplified. Naturally, evolutionists, ecologists, and organismal biologists built directly on the foundations supplied by the Modern Synthesis during this period. But just as the comparative biologists of the late 19^th ^Century could study anatomy and physiology based on a simple Darwinian foundation, so did many mid-20^th ^Century developmental and cell biologists implicitly build their research on assumptions underwritten by the Modern Synthesis: hard inheritance, no orthogenetic "direction" to evolution, adaptation by natural selection, and so on. There were prominent Western scientists who dissented from this reliance on the Modern Synthesis, like C.H. Waddington. The scientific establishment of the Soviet Union, under the direction Lysenko, also offered substantial dissent from The Modern Synthesis. But for most Western biologists, the Modern Synthesis provided a useful foundation for their research.

However, some of the assumptions at the foundation of The Modern Synthesis started to crumble in the 1970s with the discovery of super-abundant genetic variation that arguably often didn't evolve under the strict aegis of natural selection. Then cells were found to incorporate genes, mobile genetic elements, and organelles of diverse historical origins. Furthermore, it became apparent in the last decades of the 20^th ^Century that DNA sequences often evolved in ways that *reduced *the fitness of the organisms that bore them. It is now abundantly clear that living things often attain a degree of genomic complexity far beyond simple models like the "gene library" genome of the Modern Synthesis.

### Dead parts of the Modern Synthesis

It is important to be clear about common, though not necessarily universal, assumptions of mid-20^th ^Century biology that have been discarded. A partial listing would include at least the following:

• The genome is always a well-organized library of genes.

• Genes usually have single functions that have been specifically honed by powerful natural selection.

• Species are finely adjusted to their ecological circumstances due to efficient adaptive adjustment of biochemical functions.

• The durable units of evolution are species, and within them the organisms, organs, cells, and molecules, which are characteristic of the species.

• Given the adaptive nature of each organism and cell, their machinery can be modeled using principles of efficient design.

Note that some of these ideas made it easy for biologists to be specialized in their research and teaching, if not actually isolated from the concerns of other biological disciplines. Thus, before 1980, the careers of cell biologists and evolutionary biologists could proceed in relatively blithe ignorance of the concerns or findings of their distal biological disciplines. They could each insist on the purity and autonomy of their intellectual interests, cell biologists invoking their field's secure foundations in biochemistry, evolutionary biologists relying on their field's deep theoretical and mathematical heritage of population genetics, quantitative genetics, and phylogenetics.

Here we offer a general description of the emerging "new biology", and illustrate it with examples drawn from research on molecular evolution, aging, sex, and development. Naturally enough, these examples are chosen because of our own research interests. They are not intended to reflect the full sweep of the new biology, only to illustrate it. Furthermore, we do not suppose that a single review article could conceivably do justice to all the relevant complexities of research on these topics. Instead, we discuss aspects of research on these questions that serve to illustrate our general view that a new biology has developed and, in conjunction, many important assumptions of 20^th ^Century biology have been abandoned.

It might be thought that we suppose that the transition which biology is now undergoing requires the defeat or replacement of one set of biologists by another. But that is not our opinion. The senior author of this article found his way between these two kinds of biology, starting with one view of living things in 1971 and ending up with a very different one by 2001, and this was nothing unusual or creditable. We should be equally clear that, in arguing for the necessity of this intellectual transformation, we do *not *think that those who based their research on the Modern Synthesis were "bad scientists" and those who now abandon it are "good scientists." We are simply offering an overview of how a large number of us have changed our thinking, our biological Weltanschauung.

## The crucible of the new biology: molecular evolution

### The previous view of gene evolution and molecular function

In the Modern Synthesis, genes were adaptive characteristics of species, not a level of evolution with a deep history or with branching processes potentially different from those of species. This view was linked to the assumption that species history was dominated by the fine evolutionary adjustment of sub-organismal traits to specific functional ends. Strong selection capable of quickly molding traits for current utility was also expected to erase the history of sub-organismal traits. This strong commitment to the power of selection may be why Mayr wrote the following in 1963:

"Much that has been learned about gene physiology makes it evident that the search for homologous genes is quite futile except in very close relatives". – Mayr [2] p. 609

**Figure 1:**
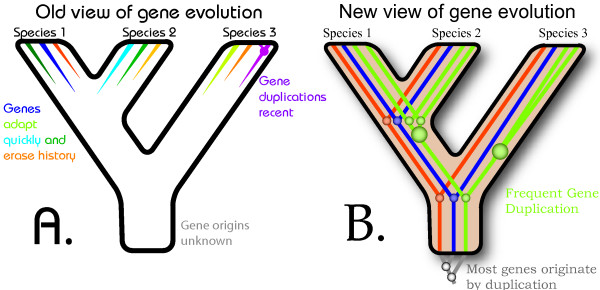
Old and new views of the evolution of genes. (A) Many 20^th ^Century biologists viewed genes as traits of species, exquisitely tuned to current utility. This resulted in the assumption that each species should, for the most part, possess different genes. Gene duplication was recognized, but was implicitly assumed to have occurred recently. (B) Many biologists now assume that most genes have their origins in gene duplication events, which happen throughout evolutionary history. As a result, many genes form families that have persisted for hundreds of millions of years.

If the genes of each species are assumed to be perfectly tuned to current function, mechanistic convergence should often result, leading not only to erasure of evolutionary history, but also to extensive homoplasy in the molecular and cellular machinery of diverse species. Thus mid-20^th ^Century biology usually assumed that species were the durable units of evolution while organs, genes, and cells evolved to match the functional demands placed on those species (Fig. 1A). When new species formed, it was expected that their genes would then diverge, and with them the cells and organs that they specified, in parallel with the opportunity for divergence that speciation supplied.

This assumption of parallelism across levels has now been widely dropped. By the start of the 21^st ^Century, molecular evolution had taught us that genes duplicate within species, and protein-coding genes are often recognizably conserved for tens or hundreds of millions of years, longer than the duration of many species (Fig. 1B).

Some of the first glimpses into the complexity of molecular evolution came in the 1960s, when the sequences of proteins from different organisms began to accumulate. Knowledge of the sequences of proteins added a new hierarchical level to be studied. No longer did proteins have to be viewed simply as characters of species. Rather amino acids themselves could now be treated as constituent characters of evolving proteins. One goal of research on molecular evolution was to use these amino acid sequences to trace species history, yet it quickly became clear that the proteins themselves had their own evolutionary histories – sometimes duplicating separately within a species with both copies persisting indefinitely.

Hemoglobin was a key molecule for the discovery of the deep history and complexity of protein evolution. Along with cytochrome C [3], hemoglobin was one of the first proteins with amino acid sequence information from multiple species [4]. Both these proteins showed deep homology across taxa separated by tens of millions of years of evolution, indicating that phylogenetic history at the gene level could now be studied on its own. In 1961, VM Ingram published a paper in *Nature *entitled "Gene evolution and the haemoglobins" [4]. Ingram presented a gene tree of hemoglobins, suggesting that the different hemoglobin chains evolved by duplication, and that myoglobin is a paralog of hemoglobins (Ingram fully articulated the concept of paralogy or "duplication-dependent homology" [5], though the word itself was not invented until 1970 [6]). Ingram recognized the importance of this idea, and considered it novel, with major implications for understanding gene evolution. Proteins have evolutionary histories of their own and deep histories at that (Fig. 1b).

### Complications for "The Tree of Life"

Nineteenth and 20^th ^Century biologists generally conceived of a "Tree of Life" – a mostly bifurcating graph connecting species in an order that reflects their common ancestry. At least three processes complicate such a view of a tree of life, horizontal transfer, symbiogenesis, and differential lineage sorting of genes. Each of these processes are at odds with fundamental assumptions of the Modern Synthesis [7,8] and a Tree of Life for the new biology is necessarily more complex than a graph joining species.

**Figure 2:**
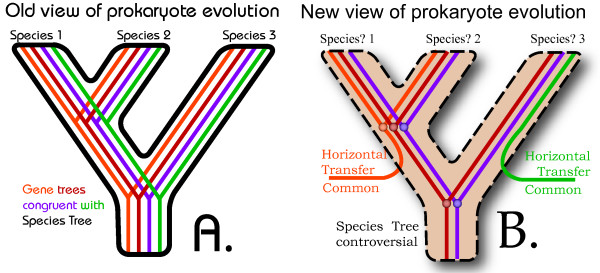
Old and new views of the evolution of prokaryote genomes. (A) 20^th ^Century biologists sometimes assumed a close congruence between gene history and species history. Horizontal gene transfer was assumed to be uncommon, as the process of genes entering a new genome is counter to the idea of a sleek and well adapted genome. (B) After analyzing the genomes of many prokaryotes, biologists recognized that horizontal gene transfer may be a common event. Furthermore, prokaryote species trees may be viewed as a patchwork of gene trees with varying levels of congruence. A similarly hierarchical view of eukaryote evolution has been articulated by Maddison [15], except that differential coalescent times – usually not horizontal transfer – is the primary mechanism used to explain incongruence of gene and species trees.

In the middle part of the 20^th ^Century, it was often supposed that organisms and their cells are sleekly functional (Fig. 2A). Given such assumptions, passing genes from one species to another would not be favorable if those genes were finely tuned for the necessary functions of the species from which they originate. Even the movement of genes within a single genome was not accepted by the biological mainstream at that time, despite McClintock's early discovery of accessory elements in maize [9]. Nevertheless, molecular characterization of transposable elements in the late 1970s finally undermined the view of the genome as a static, well-organized library of genetic information [reviewed in [10]]. With the advent of genome sequence data, researchers studying the molecular phylogenetics of bacteria realized how common prokaryotic horizontal transfer is [11,12].

Similarly, modernist preconceptions led some to discount the importance of endosymbioses in the origins of new life forms, like eukaryotes. Broad theories of endosymbiotic origins for species had been suggested in the late 19^th ^and early 20^th ^Centuries [7], but were ignored save for a few well-established cases like lichens. By the 1980s, the evidence for symbiogenesis in major cell biological events was voluminous [13,14].

Even systematics has had to abandon many strictures that were part of the Modern Synthesis. If species are the durable unit of biology, and if natural selection quickly molds genes to current utility, then most genes should diverge at the time of speciation events, given views like Mayr's. Here again, analyses of newly abundant sequence data in the late 20^th ^Century showed that rather than a highly congruent coalescence of genes at the times of speciation events, the coalescence times of alleles among species are highly variable. As such, species trees and gene trees often cannot be equated [15,16].

These phenomena complicate the tree of life. Rather than a graph connecting species, the tree of life itself is hierarchical: A universal tree of species is largely a human-imposed ideal because the components of any particular species have evolutionary histories that are not congruent with each other. This incongruence has a clear and well documented mechanistic basis in horizontal transfer, symbiogenesis and differential lineage sorting (not to mention gene duplication explained above). These processes together undermine the existence of a tree of life defined only at the level of species, pointing instead to branching histories that often differ among levels of organization and scales of analysis.

## Genomic elements of the new biology

### The periodic table and complete genome sequences

Of course it is a platitude to say that biology is an inherently hierarchical discipline. Natural science as a whole is inherently hierarchical. This is not to dismiss the existence of meaningful emergent phenomena, as the existence of life itself illustrates. But no statement in chemistry can be a false statement in physics, and no feature of life can contravene the findings of chemistry. When there is such incoherence, it has to be rectified, by correcting one or both of the conflicting disciplines.

Before the 20^th ^Century, physics and chemistry existed in partial isolation from one another. The foundations of physics were of little interest to organic chemists, just as organic chemistry was of little interest to physicists. Each could happily pursue its interests in a parochial manner. With the coming of nuclear physics and then quantum mechanics, chemistry and physics became integrated to such an extent that there is now no clear boundary between them.

Perhaps the critical bridge that links physics and chemistry is the Periodic Table. Though aspects of the Periodic Table were intuited by chemists before the 20^th ^Century, in the first half of the 20^th ^Century the Periodic Table was the obvious bridge between physics and chemistry, between the theories of quantum mechanics and the properties of chemical bonds.

In the same way, the complete sequences of genomes that were first made available circa 2000 make the interdependence of the biological disciplines patently clear. It is also evident that genomes rarely if ever are tidy libraries of biochemical instructions for making cells, nor are they the abstract assemblages of numerous alleles of small effect. Genomes clearly show the imprint of accidents in evolutionary history, selection, and biochemical constraints. Genomes are laden with mechanistic and historical detail; if not always baroque, genomes are clearly not universally elegant in their construction. And their elaborate detail implicates biochemical, cellular, organismal, ecological, and evolutionary machinery simultaneously.

### Genomics is fundamental for the new biology

The new genomic foundations of biology are not nearly as convenient as those of the Modern Synthesis:

• Genomes can have abundant DNA sequences that are of no apparent functional benefit to the organism.

• Much genomic DNA arises from the proliferation of DNA sequences that have evolved to proliferate within genomes, not benefit organisms.

• Protein-coding DNA sequences are often phylogenetically ancient, of far greater age than the species that bear them.

• Genomes can change rapidly due to selection mechanisms operating on multiple levels simultaneously, as well as processes of transposition, mutation, and recombination.

• Because the genome is a complex and shifting patchwork subject to many evolutionary and biochemical constraints and pressures, simple models of cellular or organismal function will often fail.

Common mid-20^th ^Century assumptions about how cells, organisms, and species work have thus been undermined. This might seem like warrant for despair about the future of biology, but there are two mitigations to consider. First, this complexity was always there. Darwin and many later biologists realized that their simple models were erected like piers over swampy ground. They just didn't know how deep the muck was. Second, we now have powerful genomic tools for addressing complex phenomena throughout biology. It is the use of these genomic tools in the unfolding of the new biology that we are particularly concerned with in this section of our article.

### Genomic tools of the new biology

Biologists discovered the lacunae of the Modern Synthesis through the use of some of the same tools that are already being employed to build 21^st ^Century biology. However, not all of the genomic tools that biology now uses were important in the transition to the new biology, and they might have escaped the notice of some. We start with the obvious genomic tools and proceed to those that have received less attention.

• Rapid DNA sequencing is the key technology that undermined the Modern Synthesis by revealing the complexity and variety of genomes.

• Massive parallel assays of gene expression, from mRNA production to protein level, have revealed the interconnected gene networks on which cellular and organismal functions are based. These data have undermined the 20^th ^Century notion of simple pathways of gene-enzyme determination for most biological processes, favoring instead the "network" concept of biological machinery.

• Phylogenetic bioinformatics allows us to infer the sequence changes of nucleic acids and proteins with proper statistical validity, disclosing both the unity of the biochemical machinery of life and the speed at which that machinery can evolve.

• Quantitative-genetic and genomic mapping are combining to build a genetics that can move from organism to organism with greater speed and power than the old "model organism" and "single mutant" genetics of the 20^th ^Century.

• Molecular ecology is putting DNA sequence variation and ecological processes together to increase the power of ecological research, and in so doing has revealed the high levels of complexity and species diversity, especially microbial, underlying ecological phenomena.

• Large-scale mutagenesis, RNAi and other gene expression modifications, and experimental evolution complement genomic mapping in the unraveling of gene networks, particularly by probing biological systems for their causal controls.

But there is much more going on in the transformation of biology than the mere addition of genomic technology to standard experimental strategies. The new technologies are bringing together the old disciplines of biology, from biochemistry and molecular genetics to ecology and evolutionary biology.

We will illustrate the flavor of the new biology with research on three fundamental topics: aging, sex, and development. In each of these instances, well-established 20^th ^Century views of the causal mechanisms that define each of these phenomena have been undermined, as we will now show.

## Aging: from single bullet to many-headed monster

### Cell and molecular biologists took over the study of aging inappropriately

In the latter part of the 19^th ^Century, and well into the 20^th ^Century, biologists sought to explain the phenomena of organismal aging in terms of basic organismal physiology. Around 1900, Metchnikoff [e.g. [17]] tried to explain aging in terms of autotoxification effects of the interaction between intestinal bacteria and the immune system. This theory was refuted by the persistence of aging in bacteria-free rodents. Bidder [18] tried to explain aging in terms of determinate adult body size, as opposed to the absence of aging that he assumed in fish with indeterminate, indefinite, adult body growth. Comfort [19,20] refuted this theory by showing that fish which continue growing as adults nonetheless show the increasing mortality rates characteristic of aging. And so it went with numerous other theories of aging couched in terms of organismal physiology.

With the success of molecular and cell biology in the 1950s and 1960s, molecular and cell biologists tried to explain aging in terms of general molecular mechanisms. By then it was well-established that aging was ubiquitous among species, and among organs and tissues within individual animals, particularly the better-studied mammalian organisms, like humans, rodents, and dogs. Thus Szilard [21], among others, proposed that aging resulted from an accumulation of mutations in somatic cells. Orgel [22] in turn hypothesized that aging arises from the positive feedback of errors in the translation machinery, whereby errors in the synthesis of the components of that machinery, such as amino acyl tRNA synthetases, lead to still further errors in the synthesis of those same components. These ingenious cell-level theories were complemented by Hayflick's [23,24] finding of finite cell replication among vertebrate cells allowed to divide without limit *in vitro*. For the rest of the 20^th ^Century, cell and molecular biologists largely pursued a model of aging based on the theory that limited cell proliferation leads to pervasive and cumulative failure of organismal physiology.

By the year 2000, if not earlier, it became apparent that some fundamental assumptions of this research program did not hold. Some metazoan cells, like those of fissile sea anemones and some *Hydra*, show no universal tendency to age [19,25]. Furthermore, it was realized that the molecular mechanisms which limit *in vitro *cell proliferation in humans, among other mammals, often enhance organismal survival and function by impeding the establishment and spread of malignant tumors [26-28].

In addition, despite considerable effort devoted to finding corroborative evidence for both somatic mutation and error catastrophe mechanisms of cell aging, it was generally found that cellular translation machinery tends to maintain its accuracy quite well [29] and somatic mutations were relatively limited in their damaging effects [e.g. [30]], the most significant debilitating effects of somatic mutation ironically being mutations which lead to proliferating malignancies in vertebrates [31].

After a half-century devoted to research based largely on the assumption that the cause of aging is driven by molecular mechanisms of cell breakdown, there is now abundant evidence against this characteristic assumption.

### Evolutionary biology supplied a better explanation of aging

Though most evolutionary biologists showed little interest in aging before 1980, there was nonetheless a quiet tradition of evolutionary theory devoted to the explanation of aging in terms of a progressive weakening of the force of natural selection. This line of thinking started with tangential, if not elliptical, remarks published by R.A. Fisher in 1930 [32] and J.B.S. Haldane in 1941 [33], but it was Peter Medawar who took up this theme at length, particularly in his famous 1952 essay, "An Unsolved Problem of Biology" [34]. W.D. Hamilton [35] then supplied the first mathematically cogent analysis of the age-dependent weakening of the Forces of Natural Selection. This work was placed on solid formal foundations by Brian Charlesworth, whose 1980 book [36] marked a definitive summation of this minor, primarily British, theoretical tradition.

It turned out that many of the major features of aging that were puzzling and counter-intuitive for cell biologists could be explained in terms of evolutionary theory. For example, the absence of aging in fissile organisms could be explained readily in terms of the absence of decreases in the forces of natural selection that arise when reproduction proceeds by symmetrical fission. Likewise, much of the comparative biology of aging fits readily within the framework supplied by the evolutionary theory of Hamilton and Charlesworth [31].

It was left to evolutionary experimentalists to show that aging would readily evolve as predicted by evolutionary theory [37-39]. Furthermore, it was a straightforward project to use evolutionary and quantitative genetic approaches to uncover specific physiological mechanisms that underlie aging in particular species, such as *Drosophila *[40]. Notably, the causally demonstrable mechanisms of aging in *Drosophila *have proven to be different from the molecular mechanisms assumed by cell biologists from the 1950s to the 1990s, revolving instead around resistance to stress, investment in reproduction, and metabolic reserves. Most importantly, the initial application of genomic tools has revealed that aging is a "many-headed monster" at the level of molecular machinery [41], a genomically baroque phenomenon quite unlike the well-defined, universal, aging mechanism sought by 20^th ^Century cell biologists.

### Aging is not unstoppable molecular, cellular, or organismal breakdown

But the story is further complicated as a result of additional experiments on the demography of aging. In 1992, massive experiments with dipteran cohorts showed that mortality rates did not indefinitely accelerate upward with adult age; instead, they can stabilize for what appear to be indefinite periods [42,43]. It turns out that this phenomenon is implicit in the original theory of Hamilton and Charlesworth [44,45]. Furthermore, experiments with *Drosophila *have shown that aging ceases with respect to *both *survival and fecundity, with a sustained late-life plateau of stable health that can be readily manipulated using experimental evolution [e.g. [46,47]]. Thus, many of the assumptions of 20^th ^Century research on aging no longer stand. Like many areas of biology, at the start of the 21^st ^Century, the foundations of research on aging are quite different from those assumed by the dominant paradigm of the previous century.

## Sex: from sib competition to genomic disease

### Evolutionary biologists assumed sex was a solution to ecological problems

But the preceding capsule history of the disintegration of 20^th ^Century fashions of research on aging should not be taken as a mere condemnation of the arrogance of cell biologists. We will now review a parallel set of scientific errors on the part of 20^th ^Century evolutionary biologists in which they assumed that they could explain the protean phenomenon of sex in terms of the kind of the universal, simple, causal mechanisms favored by the Modern Synthesis.

Just as cell biologists felt that it was incumbent on them to explain aging during the second half of the 20^th ^Century, Modernist evolutionary biologists had a similar notion about the explanation of sex, recombination, and kindred phenomena. And just as cell biologists naturally sought to explain what they supposed was a sustained physiological breakdown at the level of the cell in terms of the same kind of molecular mechanisms as those that they invoked to explain normal functions, evolutionary biologists naturally sought to explain sex in terms of the ecological selection pressures that had been the typical tools of evolutionary analysis ever since Charles Darwin.

Before 1970, it had been common for evolutionary geneticists to invoke simple evolutionary explanations of sex like that of H.J. Muller [48,49], who had pointed out that sexual recombination will reduce the time to fixation of beneficial mutations that arise at different loci. But by 1971, Maynard Smith, among others, realized that asexual reproduction had a two-fold advantage over sex, in that it would double the rate of production of asexual females over that of sexual females, all other things being equal [50]. Such a two-fold advantage, it was thought, would readily lead to the swamping of populations with steadily more prevalent asexual females, and the progressive elimination of sex. Furthermore, by the 1970s it was known that asexual reproduction was much more widely distributed among protists, plants, and animals than had been thought earlier in the 20^th ^Century [51,52]. By the 1980s, sex had been crowned "the Queen" of problems in evolutionary theory [53], and solutions based on 20^th ^Century evolutionary biology were certainly wanting [52,53].

A wave of evolutionary biologists set out to explain the origin and maintenance of sex in terms of the types of ecological selection pressures that were emphasized in the evolutionary biology of the 20^th ^Century. Changing environments, competition between sibs, parasite load, evolutionary arms races, and genetic load were among the range of evolutionary and ecological mechanisms that were invoked [51-54]. Some of these theories were repeated so often that, despite a general lack of critical experimental tests which corroborated them, they found their way into numerous biology texts. But none of these selection mechanisms had the reliable quality that seemed to be required for phenomena as ubiquitous as sex and recombination. Indeed, extreme and implausible ecological change seemed to be required to yield a selective advantage to sex in models based on such processes as ecological change and ecological competition. Matters were not helped by the fact that simple forms of sex and recombination were to be found even among bacteria. Sex seemed to be ubiquitous, but it was without a successful explanation based on the kinds of ecological selection mechanisms that evolutionary biologists were most comfortable with prior to 1980.

A further thorn in the side of conventional evolutionary theories of sex was that they had no credible scenario for the origin of eukaryotic sex [vid. [52]]. Under extraordinary circumstances, it was possible for evolutionary biologists to suppose that rapid ecological change or complex environments might favor the generation of abundant genetic diversity by evolutionarily long-established machinery for eukaryotic sex, but they didn't even have a cogent story to tell for the origin of sex. Yet, since eukaryotic sex is a complex and cumbersome apparatus, selection for its evolution must have been particularly intense during its origin.

### Recombination is a by-product of normal DNA repair mechanisms

The suspicion that evolutionary biologists were "barking up the wrong tree" started to grow among biologists in the 1980s. One of the important points brought to the debate was the intimate relationship between the molecular machinery of recombination and that of DNA repair. DNA repair is one of the most fundamental needs of all organisms. It became apparent that a side-effect of double-strand break repair in cells with homologous chromosomes might be recombination [54-56]. Given this fact, why did there have to be some type of ecological selection for recombination? Selection for the maintenance of the chromosome would suffice. As of the first years of the 21^st ^Century, evidence that chromosomal recombination is a by-product of DNA repair has continued to grow [57,58]. This is not to say that we are arguing the evolution of sex is *only *determined by selection for DNA repair. Rather, we are making the point that biologists are now considering a much greater diversity of evolutionary mechanisms for sex, including mechanisms at multiple levels of the biological hierarchy.

### Sex may have originated and be maintained as a form of genomic parasitism

Growing information about transposable elements made it clear by 1980 that some DNA molecules could copy and spread among genomes as a result of selection on such DNAs to spread within and among genomes, not as a result of selection between organisms [59,60]. Furthermore, the spread of such parasitic elements depends critically on the occurrence of sexual recombination and horizontal gene transfer. So long as cells and organisms did not recombine DNA with each other, transposable elements would be selected to control their proliferation within genomes [61].

This paved the way for the proposal that sex originated as a device for parasitic DNAs to spread from cell to cell [61-63]. Experiments with autonomous conjugative elements with deleterious effects [64-66] showed that such parasitic elements could spread *de novo *in bacteria, making it plausible that ancestral unicellular eukaryotes may have evolved sex by analogous means.

The problem of the maintenance of sex also was attacked from the vantage point that sex didn't have to be beneficial to be maintained. It was shown mathematically that, when potentially asexual females suffer from continued fertilization by males, anisogamous sex could be maintained even if it was not evolutionarily beneficial in itself [67]. Recently, the natural history of sex has been interpreted in terms of the ability of new parthenogens to avoid sex with males [68]. It is a notably inelegant feature of this research that it brings in genomic detail as well as historical effects in the analysis of the evolution of sex.

### Sex has been subject to natural selection at multiple levels

As the 20^th ^Century waned, it was becoming apparent that many aspects of the evolution of sex and recombination were more suited to explanations couched in terms of transposable elements, chromosomes, and organelle genomes undergoing selection *within *organisms, rather than the radical and implausible processes of ecological change assumed by the evolutionary explanations of sex based on standard models derived from the assumptions of the Modern Synthesis. This did not show that the evolution of sex was *never *shaped by ecological factors. Indeed, a miscellany of experimental data accumulated suggesting the occasional role of ecological factors in the evolution of sex. What was rendered otiose was the sole reliance on ecological selection as the explanation for the maintenance of sex and recombination.

The study of the evolution of genetic systems now characteristically involves the invocation of multiple levels of selection, between and within genomes, and diverse molecular processes, from transposition to unequal chromosome segregation during gametogenesis, and so on [69-72]. Evolutionary ecology models for the evolution of sex have been put in their proper perspective, as some among many of the mechanisms that contribute to the scientific complexity of sex. With respect to its eclecticism, the study of the evolution of genetic systems has become a promising portent for the future of biology as a whole in this century.

## Homology and evo-devo: ancient genetic toolkits for development

### Ancient Gene Histories are Widespread

In the 1980s and 1990s, biologists began discovering deep homologies in body patterning genes like *Hox *genes [73,74]. The view that many derived from the Modern Synthesis had been that organismal structures like segmented bodies, eyes, limbs, and hearts, evolved essentially *de novo*, multiple times, independently in various lineages, in close conformity with the requirements of function. Like the concept of genes in the days preceding the advent of modern genomics, the common assumption was that organismal structures were simply traits of species. Species might have a particular morphological trait for a particular function, or they might not have that trait if that function was not selectively favored. Few paid attention to the intermediate possibility – that morphological traits themselves are a complex patchwork of shared and derived elements, and thus are more analogous to baroque ornamentation [72]. For example, 20^th ^Century biologists often assumed that distinct developmental processes often arose separately in different lineages – especially when comparing different phyla, which were regarded as having different "body plans". But the discovery of conserved developmental genetic processes for patterning the bodies of taxonomically and morphologically disparate organisms forced biologists to consider common descent at deeper levels of biological organization.

### Eye evolution uses common ancestral genetic components

Eye evolution provides a canonical example. A comprehensive, and oft-cited, 1977 publication [75] concluded that photoreceptors must have evolved essentially *de novo *40–65 times independently. This is an excellent example of the then-common emphasis on selection over evolutionary history, just before this view was to collapse.

Research in the 1990s showed that many of the genes involved in eye development are homologous between phyla. Although it had been known for some time that the opsin visual-pigment genes are conserved across phyla, a watershed discovery in eye evolution was that similar mutant eye phenotypes in fruit flies, mice, and humans are caused by mutations in homologous genes [76]. These genes are named *Pax-6 *in vertebrates and *eyeless *in fruit flies. Multiple other phyla are now known to utilize homologous *Pax-6 *proteins during eye development [77]. Furthermore, multiple genes besides *Pax-6 *and *opsin *were subsequently shown to have a conserved role in eye development across phyla. Nonetheless, other components of animal eyes, like lens proteins, have in many cases only relatively recently been co-opted for use in eyes [78]. As such, disentangling questions like "Are all eyes homologous?" is complex. The answer invariably is "some parts of eyes are homologous and some parts are not" and the answer also depends on the time scale being examined.

### Evo-Devo has been discovering common underlying genetic toolkits

Similar stories to that of eyes can be told for limbs, hearts, and body segments. Naturally, many biologists now question the assumption that organs and especially developmental processes normally evolve multiple times from scratch, each specifically fashioned by powerful mechanisms of natural selection. Present-day biology emphasizes the importance of deep homology among developmental processes and the genes that code for them, and also the recombination of these elements to form novelties. As such, developmental processes are far from elegantly constructed, being instead the result of bricolage or tinkering [79,80] as well as duplication and divergence at multiple biological levels, thus making evolutionary history of central importance for understanding organismal traits [81].

## New rules for biology

While the nature of the transition from 20^th ^Century biology to 21^st ^Century biology seems clear to us in both overview and for some particular applications, the most important changes defining this transition might be usefully listed:

• We should no longer assume that a biological research problem can be satisfactorily solved using the intellectual tools from only one biological discipline. This might be the case, but it is likely that most valid one-discipline solutions are the 'low-hanging fruit' already picked by 20^th ^Century biology.

• We cannot assume in advance the existence, level, or focus of natural selection on a particular biological attribute. The attribute could arise from (i) accidental evolutionary events, (ii) selection on a DNA sequence that evolves independently of the replication of its host, or (iii) unanticipated pleiotropic effects of selection on other characters.

• We cannot assume fixed relationships between structures and functions. Most evolutionary histories are complex, with structures adopting different roles in the course of their evolution, and functions being underlain by different structures during the course of their evolution. That is, the causal hierarchies of biology are not necessarily fixed, even in overall structure.

• We cannot assume the stability or distinctness of rate among biological processes. Genetic evolution may occur on a comparable time-scale to that of ecological change, for example.

• Unlike stereotyped scientific practice, 21^st ^Century genomic technologies and techniques of data analysis give us the opportunity to be "led" by the data in an hypothesis-free manner. When appropriate, we may "listen" to the data, rather than forcing a particular hypothesis on it.

• In the same spirit, experimental evolution and experimental ecology let organisms show us how they respond to particular biological regimes, which is complementary to performing critical experimental tests of a priori hypotheses.

• Numerical exploration of theoretical models for complex biological mechanisms will be more informative than assuming away most of the biology in order to achieve a mathematically-refined global analysis. Again, like genomics and experimental evolution, such numerical work can be exploratory and open-ended, rather than seeking a pre-determined outcome. The low cost of computation makes such open-minded numerical work vastly more feasible.

• Modern statistical and bioinformatic techniques are likewise considerably more powerful, which allows us to collect and analyze information on a vast scale. These data allow a more thorough exploration of the complexities of biology.

Some may feel that the view of life supplied by nascent 21^st ^Century biology is painfully complicated, if not perverse. For our part, we think that the historical complexity and versatility that we now know to characterize life are inspiring and challenging. In many ways, we are reminded of the transition from the complacent physics of the 19^th ^Century to the turbulent modern physics after Einstein's 1905 scientific revolution. The old Newtonian certainties were destroyed, but in their place physicists found both a better foundation for their field and a spectrum of exciting research problems. We feel that biology has found its way to the same level of maturity.

## Nothing new?

Some readers may feel that much that we have said is simply stating the obvious. But we are not proposing the creation of a new biology. It already exists, and it is the only biology that many present-day graduate students have known. Yet it is always useful to know the country that you reside in, particularly for those of us who are older and who started our scientific careers at another point in the historical development of biology.

Others might assert that we are describing nothing more than systems biology. But there are major historical problems with this view, namely, the field of systems biology itself has undergone the same sort of transformation as other biological disciplines mentioned previously. Systems biology was invented at the same time as the Modern Synthesis, explicitly by Ludwig von Bertalanffy and in many respects also implicitly by Sewall Wright, in the 1930s. To give just one example, von Bertalanffy published his much-read book *General System Theory *in 1968 [82]. Both of these titanic figures were interested in complex networks of biological regulation and organisms as dynamical systems, yet these ideas were hardly incompatible with the then-prevailing Modern Synthesis in content. Examination of the contemporary literature of systems biology show that it involves core ideas that have been common among devotees of the thinking of Bertalanffy and Wright, and also Odum, Rapoport and other practitioners of systems thinking in biology from the 1930s to the 1970s, covering biological sub-disciplines from organismal biology to ecosystem modeling. But modern systems biology, just like the broader field of biology, also includes further articulation and elaboration of ideas, many of which are incompatible with assumptions of the Modern Synthesis.

But a more constructive answer to a criticism that today's biology is not fundamentally different is to give a concrete example. Take "the C-value paradox," the fact that total eukaryotic genome size, in number of nucleotides, is sometimes vastly greater than the size required to code for the complete set of genes and regulatory sequences of any known eukaryote species. The reason this observation is named a paradox at all relies on the assumption that genomic complexity should be correlated with organismal complexity. Why should the genomes of some ciliates, salamanders, and ferns be vastly larger than those of humans? From the perspective of the Modern Synthesis, one might suppose that these ciliates, salamanders, and ferns have much greater need for numerous, diverse, structural genes or regulatory sequences.

Present-day biologists are instead dealing with the C-value paradox in terms of the evolution of non-coding DNA, the proliferation of transposable elements, and kindred phenomena. This does *not *imply that evolutionary theory simply no longer applies. Instead, new evolutionary theories have been developed, such as Lynch and Conery's theory of reduced effective population size leading to less efficient selection against the proliferation of non-essential, even deleterious, DNA sequences, and thus greatly expanded genome sizes in endemic species with small population sizes [83,84]. It is not our concern to argue for or against this particular theory, only to point out that this scientific debate was not a live issue for biologists in 1970. And it certainly wasn't an obvious corollary of the systems biology perspective then extant, either.

The fundamental landscape of biology is undergoing a major upheaval, much as it did in the first decades of the 20^th ^Century [1]. This upheaval will take time to fully reveal its implications. The sequencing of several important eukaryotic genomes around the year 2000 was no more an instant transformation of biology than the re-discovery of Mendel was in 1900. Decades are required to change the foundations of a scientific field as complex as biology. Furthermore, the new biology is not without its anticipatory prophets, and we do indeed consider Barbara McClintock, Sewall Wright, Ludwig von Bertalanffy, C.H. Waddington, Emile Zuckerkandl and others to be such figures. But biological research is undergoing a period of rapid, and profoundly beneficial, transformation reminiscent of the events surrounding the Modern Synthesis. We further hope that biology curricula and textbooks will eventually come to reflect this disciplinary transformation, much as the teaching of biology was reformed to reflect the Modern Synthesis during the middle part of the 20^th ^Century.

## Competing interests

The author(s) declare that they have no competing interests.

## Authors' contributions

MRR prepared the first draft of this article, supplying the overall theme and developing the examples of research on aging and sex. THO then added the material on molecular evolution, phylogenetics and "evo devo," as well as contributing the figures. Both authors then worked on all parts of the manuscript through its successive revisions, reading and approving the final manuscript.

## Reviewers' comments

W. Ford Doolittle, Dalhousie University, Halifax, Nova Scotia, CANADA:

Providing comments on the submission by Rose and Oakley is a pleasure, a challenge and (on another level) a source of some irritation. *Comments*, not really a review, because this essay, like many published in Biology Direct is an historically-grounded speculative opinion-piece. It cannot really be judged as valid or invalid in the same objective way that we like to believe that experiments and the interpretation of experimental results can be. For fair-minded reviewers, rejection is not a serious option. A *pleasure *because the grand science history story Rose and Oakley tell is congruent enough with my own version of collective experience that I can endorse it, and it is very well articulated. A *challenge *because I don't think there is in fact *any way at all *that such metanarratives can be judged to be valid or not, no matter who tells them. Each of us comes to believe what he/she does about the status of his/her discipline through an idiosyncratic mix of personal experience, interactions with colleagues, the primary literature and whiggish "histories" written by others in that discipline. Of course there has also long been, especially in evolutionary biology, a grander tradition of synthetic theory, several of whose champions Rose and Oakley cite, and in which tradition their own ms will no doubt find a solid place. To the extent that such theory-building self-consciously situates itself within comprehensive historical accounts constructed to make the theory look good (a practice of which anyone who operates at this level is guilty), it is suspect. But not useless: we need shared stories no less than any other community. And *a cause for irritation *because I want to do a proper job on something like this, especially since my words will be published. But a proper job can use up as much creative energy as writing my own historically-grounded speculative opinion-piece, and the effort will not even be picked up by PubMed: you'll have to look on Google. I suggest that Eugene send reviewers of Biology Direct articles who contribute more than, say, 1,000 words, T-shirts emblazoned "I reviewed a manuscript for Biology Direct and all I got was this lousy T-shirt". People could wear them at meetings.

This churlishness aside, let me disagree with Rose and Oakley about the numbering and naming of "biological syntheses" or re-integration events. Darwin's and the Modern Synthesis were indeed the first and second but pre-genomic molecular biology was as complete a re-integration (let's call it the Molecular Synthesis). My evidence for that are the legions of successfully practicing molecular biologists who never read a book by George Gaylord Simpson or Ernst Mayr, although to be sure molecular biologists until recently would have enthusiastically endorsed the five "dead parts" of the Modern Synthesis that Rose and Oakley bullet. I especially agree with them that at the outset molecular biologists were hard-core adaptationists, and see for the first time thanks to them how this connects to our early expectation that although what was true for *E. coli *might be true for the elephant, we would not find homology at the level of genes, detectable in sequences. My fourth re-integration would be the Genomics Synthesis, which now, as "systems biology", promises top-down answers to questions of cellular function. Claims that we need and can have a truly holistic biology have been around for a long time. Maybe now they will be fulfilled, although I do worry about the loss of historical perspective that viewing cells and organisms as integrated systems seems to entail: we are at risk of forgetting that evolution is a tinkerer. And my fifth would be the Metagenomics Revolution, which for microbiologists should be enormously constructive in a deconstructive way – enabling us to get rid of the belief that organisms must be clustered in species, or that we can speak meaningfully about the ancient history of modern "lineages".

So maybe actually I see Rose and Oakley's New Biology as a three-step revolution culminating in what we might call the Postmodern Synthesis, in which we at last rid ourselves of (at least) two deeply-embedded and probably theism-based pre-Darwinian notions: that species and higher taxa are real (what Mayr called typological thinking), and that organisms are as perfectly ("sleekly" in Rose and Oakley's apt phrase) functional as watches, although natural selection and not God is the perfect watchmaker. We're not there yet, as debates over the Tree of Life and a rash of new papers on the functions of junk DNA reveal. In this sense I think Rose and Oakley, perhaps because they are evolutionists, are too generous to the many molecular systematists who believe that they can put Bergey's Manual of Systematic Bacteriology on a solid footing with rRNA phylogenies and to the many molecular biologists who still mostly concern themselves with how organisms work rather than why, and assume an optimality which much of the data speak loudly against.

Each of the topics Rose and Oakley discuss in detail drives home the same point: we start out thinking that what seems to be a common biological pattern should have a common causal process-level explanation, and wind up discovering a variety of processes, undermining the reality of pattern. Sometimes evolutionary theory shows us why a certain outcome (aging, multilevel selection) might be inevitable, but seldom does it tell us how or how often this end has been achieved. What we get instead is an explanatory toolkit to apply to specific cases, making the present comprehensible in terms of the past. Such explanatory pluralism defines the Postmodern Synthesis. I see this as the final maturation of biology, a discipline more akin to history than to physics, properly viewed. I don't think our end-state is "systems biology", if in using this term we hope for some grand unifying theory of biology. I don't think there can be such a theory any more than there can be one for human history. Perhaps Rose and Oakley can commit themselves on this.

That's my thousand words: where's my T-shirt?

*Authors' Response: We are very grateful to have this entertaining and interesting review from Ford Doolittle, who has done so much to bring the biological research community into the brave new world of post-Modernist biology*.

*We will comment (agreeing with him that such opinions cannot be judged) on two of the major points raised by Dr. Doolittle. First, he outlines five synthesis or re-integration events. One of these is his "Molecular Synthesis". We are well aware of the degree to which some molecular biologists disdained contact with the intellectual life of modern evolutionary biology, beyond a few patronizing and often scientifically inaccurate allusions to Charles Darwin. For this reason, we do not see this period as a time of synthesis. Although an undeniably important time in the history of biology, the attitudes cited, coupled with the new found reductionist power of molecular biology, tended to splinter, not integrate biology. Second, Dr. Doolittle raises the possibility that biology has no overarching unifying theory, and that a mature explanatory toolkit is the apex of biology. Without a crystal ball, and given the complexity and hierarchical nature of biology, it is difficult to argue against this idea*.

Eugene V. Koonin, National Center for Biotechnology Information, National Library of Medicine, National Institutes of Health, Bethesda, MD:

This is an ambitious, even super-ambitious paper. The subject is, no more, no less, the change of the very character of modern biology that, according to Rose and Oakley, has taken place (or, perhaps, is still taking place) at the end of the 20^th ^and the beginning of the 21^st ^centuries. I think the authors are, generally, correct in their claim that such a crucial transformation of biology, indeed, has occurred. Many biologists, of course, intuit this change but few seem to rationalize it. Therefore, this is, potentially, a paper of unusual importance, perhaps, an eye opener of sorts to many.

So what are the key differences between the "new biology" and the traditional one that reigned in the 20^th ^century and, to a significant extent, is epitomized by the Modern Synthesis of Evolutionary Biology? In one word, the name of the new game is plurality. To use, perhaps, a somewhat tacky phrase, the unity of the modern synthesis has been replaced by a postmodern disarray (postmodernism is not mentioned in the current version of the article but I feel the notion just begs to be let in, with all necessary qualifications).

***Author's Response: ****Dr. Koonin's point is well taken that the "New Biology" which we have described fits many definitions of postmodernism, though certainly not all. In fact, a previous version of this manuscript was entitled "Post-modern Biology", with references to postmodernism throughout. Although we agree with Dr. Koonin about these parallels, we decided that explicitly including postmodernism raises too many issues of semantics, if not indeed hermeneutics. To do justice to the concept of postmodernism, especially for an audience of biologists who may or may not be familiar with the intricacies of the concept of postmodernism, would require extensive discussion of a non-biological topic. Without such an extensive discussion, using the term postmodern easily could be taken as simply a pun, describing the state of biology after the Modern Synthesis. Already, we could not cover all biological topics in sufficient detail in a relatively brief article (see below)*.

This is the emphasis of the article but herein, as I see it, also lie some limitations of Rose's and Oakley's text. I fully realize that the subject is vast and complex, and cannot be humanly covered in its entirety in a relatively brief article, so selections must be made, illustrative examples have to be used etc. This being said, I believe that there are several areas of substantial, structural incompleteness, so I try to briefly discuss these below.

1. The most important shortcoming, to me, is that this manifest of the postmodern, pluralistic biology, actually, is very modern or even classical in a crucial respect, its eukaryote-centrism; the exception is the brief discussion of the crucial role of horizontal gene transfer in the evolution of prokaryotes (I come back to this issue below). All the illustrations and most of the discussion revolve around biological issues that are specific to eukaryotes such as sex, development, and aging. Of course, it is not unreasonable for the authors to address problems that are closest to their own areas of expertise but the importance of abandoning the eukaryote-centrism and, with it, any persisting notion of progress in the evolution of life must be made crystal clear. Indeed, one of the essential dimensions of the newly realized pluralism in biology is the fundamental plurality of life forms and the corresponding, astonishing diversity of genome organizations, replication-expression strategies, cellular organizations, and lifestyles. Prokaryotes were not at all considered within the framework of the Modern Synthesis, and viruses were not even viewed as a major class of biological entities. The discoveries of the last third of the 20^th ^century, in particular, those of molecular phylogenetics, have changed these perceptions forever. We now realize that the lowly "monera" actually comprise two of the three domains of cellular life (bacteria and archaea), harbor most of life's metabolic diversity, and comprise most of the cellular biomass on earth. Add to this that viruses are, in all likelihood, the most common biological agents on earth (the number of viral particles in many habitats exceeds the number of cells by about an order of magnitude [85,86] and that, unlike cellular life forms that follow the Central Dogma, viruses display, essentially, all imaginable strategies of replication and expression [87,88]. The realization that archaea, bacteria, and viruses are parts of life that are every bit as fundamental as eukaryotes – quite possibly, more fundamental inasmuch as eukaryotes very well might be hybrids of archaea and bacteria [89,90] – has profound consequences for the reappraisal of the Modern Synthesis and the tenets of New Biology discussed in this article. Let us take a look at the "dead" parts of Modern Synthesis, according to Rose and Oakley and reassess the status of each postulate in view of the true diversity of life forms:

(i) "The genome is a well-organized library of genes"

*-*It is debatable just how well organized prokaryotic genomes are but, unless optimality is seriously claimed, they do appear to be reasonably good libraries.

(ii) "Genes usually have single functions that have been specifically honed by powerful natural selection"

-Indeed, this does not seem to be the case in any life forms.

(iii) "Species are finely adjusted ..."

-Not just dead but, simply, makes no sense as generalizations because species cannot be objectively defined in prokaryotes and viruses, and however they might be defined arbitrarily, cannot possibly be the fundamental units of anything.

(iv) "The durable units of evolution are species..."

-Makes no real sense – see above.

(v) "Given the adaptive nature of each organism and cell, their machinery can be modeled using principles of efficient design"

-Prokaryotes are much more streamlined machines than eukaryotes; while not every aspect of their functioning can be modeled using design principles, such modeling is generally not considered hopeless.

Now to the "genomic foundations for the new biology":

(i) "Genomes can have abundant DNA sequences that are of no apparent functional benefit to the organism"

-Not really abundant when it comes to prokaryotes and, especially, viruses.

(ii) "Much genomic DNA arises from the proliferation of DNA sequences that have evolved to proliferate within genomes, not benefit organisms."

-Not that much in prokaryotes, and virtually, none in viruses (and many of the viruses do not even have any DNA at all); those elements are under tight control in most if not all prokaryotes, and in most unicellular eukaryotes as well.

(iii) "Protein-coding DNA sequences are often phylogenetically ancient, of far greater age than the species that bear them..."

-This is, indeed, universally true. Many proteins (and structural RNAs as well) are conserved throughout the entire history of life as we know it (not just older than species that cannot even be meaningfully defined in prokaryotes and viruses).

(iv) "Genomes can change rapidly due to selection mechanisms operating on multiple levels simultaneously, as well as processes of transposition, mutation, and recombination."

-These rapid changes are even much more dramatic in prokaryotes and viruses than they are in eukaryotes.

(v) "Because the genome is a complex and shifting patchwork subject to many evolutionary and biochemical constraints and pressures, simple models of cellular or organismal function will often fail."

-Yes, simple models will fail often, but the extent of the failure depends on the complexity of the modeled system, i.e., for a simple prokaryotic cell, or a virus propagating within a cell, the chances of some level of success are much greater than for a complex eukaryotic cell.

Thus, the consideration of the full spectrum of life forms sends a message that, I think, is even more powerful than, simply, deconstruction of the Modern Synthesis. The inevitable conclusion from comparative-genomic analyses is that the prevailing forces affecting genome evolution in prokaryotes (and viruses) and in eukaryotes, and the resulting genomic layouts are dramatically different. They also substantially differ among different bacteria, even more so, between unicellular and multicellular eukaryotes, and even within the latter, say, between different animals. Plurality of pattern and process rules supreme, and the challenge is to uncover the underlying logic of evolution – if any such exists.

***Authors response ***– *We thank Dr. Koonin for expressing his prokaryote-centered views quite forcefully. We agree that his viewpoint underscores even further the idea that a pluralistic new biology is upon us. Despite the good fit of this view point, we decided in the main article to keep our original intention of focusing primarily on our own specialties when discussing examples of the new biology. Dr. Koonin's review provides an outstanding supplement to our article, focused on one of his own specialties*.

2. Rose and Oakley bring up the newly apparent prevalence of horizontal gene transfer as one of the major blows to the 20^th ^century perspective in biology. This is, certainly, true, but I think the discussion in the paper stops short of really driving the nail down. The real issue is that, when fully conceptualized, extensive HGT undermines the very notion of the Tree of Life (the TOL paradigm) which, certainly, is a big part of the Modern Synthesis (as well as the classical, Darwinian foundation of biology). Simply put, although trees are crucial in depicting certain phases and aspects of life's history, there is no TOL as such, i.e, evolution of life cannot be presented as a tree, so Darwin's famous simile fails as an overarching generalization. The demise of the TOL paradigm is covered in several recent papers [91,92]. Again, this is related to the problem of "eukaryotic chauvinism": the tree pattern might hold for the evolution of the major divisions of eukaryotes (although not necessarily for all eukaryotes taken together) but, certainly, not for prokaryotes, let alone the entire history of life.

***Authors response ***– *Here we disagree with Dr. Koonin. HGT does not necessarily undermine Darwin's "Tree of Life" completely, even though in post-Modernist biology this Tree of Life is much more complex. Today's Tree of Life, as Dr. Koonin points out, is different from what Darwin envisaged, in that it is multi-dimensional – branching histories characterize in a complex way multiple levels of organization, not just the species level. Further, as discussed in the article, HGT is not the only blow to a two-dimensional tree; paralogy, endosymbiosis and lineage sorting also contribute to a new, highly multi-dimensional view of evolutionary history*.

*This emerging understanding of the trees of life is pluralistic, encompassing the branching history of biological units at all different levels of organization *[81]. *The evolutionary histories of units at different levels (gene domains, genes, species, etc) are not always congruent with each other, yet there are still branching histories that characterize each of these levels. Branching history is a pattern that results from well known mechanisms including exon shuffling, gene duplication, genome duplication (polyploidy), co-option, speciation, and vicariance of multiple species. HGT is one example of a mechanism that causes branching histories at different levels of organization to be incongruent. It clearly points out the failed assumption that the history of components is congruent with the history of the higher level unit to which it belongs. Nevertheless, this assumption can be used as a valuable null model to understand macroevolutionary patterns and processes *[93]. *As we discussed in this article, the species was usually seen as the durable unit driving branching at all levels, but the existence of multiple evolutionary levels and mechanisms violates this assumption*.

*Processes to split biological units pervade all levels of the biological hierarchy. Protein domains duplicate within genomes and may be "horizontally transferred" from one gene to another. Genes may form units of synteny or operons, but individual genes may also be copied from one part of the genome to another or from one genome to another, independently of the rest of a synteny unit or operon. Whole chromosomes and whole genomes may also duplicate by various mechanisms. All these processes create the new tree of life. But that tree is a postmodern tree, rich in complexity. Components coalesce to form units with a congruent path for a time, only to be broken up. There is no reason to provide anti-intellectual, anti-evolutionists with quotes like "The Darwinian paradigm is dead", because this complexity only enhances Darwin's most profound insight – the universal common ancestry of life*.

3. It appears that, to a very large extent, Rose and Oakley identify the transition from modernism to post-modernism in biology with the fall of the pan-adaptationist paradigm of the Modern Synthesis. I believe this is, indeed, a sensible view, but if so, the conceptual/intellectual history of this change is not quite adequately presented in the paper. To me, the (near) neutral theory of molecular evolution (Kimura, Jukes-King, Ohta, and more recent developments) is a major and indispensable part of the story. The intellectual importance and impact of Jacob's tinkering paper and, especially, Gould and Lewontin's spandrel paper cannot be underestimated either. These landmark papers are cited in the present article but, I believe, not entirely in the right context. The name of Richard Dawkins does not come up at all although the conceptual significance of *The Selfish Gene *for our current understanding of different level of selection and the prominence of selfish elements is, in my opinion, impossible to deny. In the same vein, it is hard to envisage the evolution of the postmodern view of genomic evolution without the historic papers of Sapienza-Doolittle [59] and Crick and Orgel [60] on selfish DNA. In my opinion, another important part that, in a sense, wraps up the debunking of the Panglossian view of life's evolution [see [94]], comes in the recent papers of Lynch on the neutralist concept of the origin of genomic complexity [83,84] – again, cited here but, I am afraid, not entirely in focus.

*Author's response – We have added these important references*.

4. Finally, a few words about possible glimpses of a forthcoming new synthesis. The comparison to quantum/relativistic revolution in physics is, I think, fully appropriate. The problem is, however, that we do not have a clear image of the new synthesis in biology. "Postmodern synthesis" is an oxymoron of sorts as post-modernism eschews (more or less) all generalization. However, this flies in the face of the way we do science. We need a meta-narrative in order to move on, even as we realize, clearer than ever, that it should not be construed as anything even approaching the "true" representation of biology, only as a worldview that is not as obviously and hopelessly flawed as the previously prevailing vision. I believe that some aspects of the new worldview have already been developed, with relatively little fanfare. The work of Lynch quoted above establishes a neutralist null hypothesis for the evolution of genomic complexity, rooted in the specifics of the population structures and dynamics of different organisms, and in that capacity it can be regarded as the logical completion of the post-modernist transformation of biology. I am far from suggesting that this concept provides the single underlying principle of biological evolution (Rose and Oakley convincingly argue that no such thing is attainable) but it is one of the crucial parts of the new framework. In addition, it is hard for me to pass "evolutionary systems biology" [e.g. [95,96]]. I believe that the efforts to understand and integrate the multiple inputs that affect a gene's evolution (e.g., expression level and position in various types of networks) fit well into the pluralistic foundation of new biology, and might be pointing to some of the important facets of the new biological mainstream.

A very final, conceptually minor but not altogether unimportant comment. I think mentioning Lysenko and his henchmen as scientific opponents to the Modern Synthesis (and in the same breath with C. H. Waddington, a genuinely outstanding biologist) is, to put in mildly, disingenuous. Lysenko et al. were criminals driven by political motivation of the worst kind, not scientists.

John M. Logsdon, Jr., Roy J. Carver Center for Comparative Genomics, University of Iowa, Iowa City, IA, USA:

This reviewer expressed approval for the publication of the article without further comments.
